# Automatic Segmentation of the Cisternal Segment of Trigeminal Nerve on MRI Using Deep Learning

**DOI:** 10.1155/ijbi/6694599

**Published:** 2025-02-16

**Authors:** Li-Ming Hsu, Shuai Wang, Sheng-Wei Chang, Yu-Li Lee, Jen-Tsung Yang, Ching-Po Lin, Yuan-Hsiung Tsai

**Affiliations:** ^1^Center for Animal Magnetic Resonance Imaging, The University of North Carolina at Chapel Hill, Chapel Hill, North Carolina, USA; ^2^Department of Radiology, The University of North Carolina at Chapel Hill, Chapel Hill, North Carolina, USA; ^3^School of Cyberspace, Hangzhou Dianzi University, Hangzhou, China; ^4^Department of Diagnostic Radiology, Chang Gung Memorial Hospital, Chiayi, Chiayi, Taiwan; ^5^College of Medicine, Chang Gung University, Taoyuan, Taiwan; ^6^Department of Neurosurgery, Chang Gung Memorial Hospital, Chiayi, Chiayi, Taiwan; ^7^Institute of Neuroscience, National Yang Ming Chiao Tung University, Taipei, Taiwan; ^8^Department of Education and Research, Taipei City Hospital, Taipei, Taiwan

## Abstract

**Purpose:** Accurate segmentation of the cisternal segment of the trigeminal nerve plays a critical role in identifying and treating different trigeminal nerve–related disorders, including trigeminal neuralgia (TN). However, the current manual segmentation process is prone to interobserver variability and consumes a significant amount of time. To overcome this challenge, we propose a deep learning–based approach, U-Net, that automatically segments the cisternal segment of the trigeminal nerve.

**Methods:** To evaluate the efficacy of our proposed approach, the U-Net model was trained and validated on healthy control images and tested in on a separate dataset of TN patients. The methods such as Dice, Jaccard, positive predictive value (PPV), sensitivity (SEN), center-of-mass distance (CMD), and Hausdorff distance were used to assess segmentation performance.

**Results:** Our approach achieved high accuracy in segmenting the cisternal segment of the trigeminal nerve, demonstrating robust performance and comparable results to those obtained by participating radiologists.

**Conclusion:** The proposed deep learning–based approach, U-Net, shows promise in improving the accuracy and efficiency of segmenting the cisternal segment of the trigeminal nerve. To the best of our knowledge, this is the first fully automated segmentation method for the trigeminal nerve in anatomic MRI, and it has the potential to aid in the diagnosis and treatment of various trigeminal nerve–related disorders, such as TN.

## 1. Introduction

The trigeminal nerve, the largest among the cranial nerves, plays a vital role in facilitating touch, pain, and temperature sensation in the face. Trigeminal neuralgia (TN) is a prevalent facial pain disorder characterized by recurrent, brief, and paroxysmal episodes of pain. Individuals with TN often describe the pain as stabbing or resembling an electric shock, primarily localized to the sensory distribution area of the trigeminal nerve. Neurovascular compression at the root entry zone stands as the primary cause of idiopathic TN, accounting for the majority of cases [1], and classical TN is characterized by its development without an apparent cause, except for neurovascular compression [2]. Trigeminal neuropathy, on the other hand, presents a similar clinical profile but differs in its etiology, resulting from nerve lesions or trauma and frequently accompanied by sensory neurological deficits.

Although clinical symptoms are used for diagnosis, neuroimaging, especially magnetic resonance imaging (MRI), is also important for differential diagnosis and ruling out conditions such as tumors, arachnoid cysts, and vascular anomalies. High-resolution MRI advances further enable the evaluation of the trigeminal nerve's condition for TN diagnosis and treatment. This includes determining the site and severity of neurovascular contact, as well as identifying any displacement or atrophy of the trigeminal nerve and the involved vasculatures [1]. This information significantly enhances the accuracy of microvascular decompression surgery [3], imaging-guided nerve ablation [4], and radiosurgery [5]. However, the inconsistent results of manual segmentation and the unclear causal relationship between neurovascular contact and TN have contributed to significant uncertainty regarding the utility of this approach, despite the advancements made in the field [6].

Traditional methods of segmenting the trigeminal nerve from MRI have relied on manual tracing by radiologists or neurologists, which is time-consuming, is prone to human error, and can be subjective. On the other hand, deep learning, a subset of artificial intelligence, has shown great promise in medical imaging, where it has the potential to improve the accuracy and efficiency of diagnosis, treatment, and patient outcomes [7]. Deep learning algorithms can be trained on large datasets of medical images to detect subtle abnormalities, segment structures, classify images, and predict outcomes. The use of deep learning in medical imaging can also help reduce the workload of radiologists and healthcare professionals, allowing them to focus on other aspects of patient care [8]. However, there have been limited applications of deep learning in the segmentation of cranial nerves thus far. The aim of this study is to develop an automatic segmentation model for the cisternal segment of trigeminal nerve in structural MRI for patients with TN by deep learning.

## 2. Materials and Methods

### 2.1. Dataset

This prospective study was designed to use the brain MRI scans of both health controls (HCs) and TN patients. Two disjoint parts of MRI data were collected in this study. One part of data was used for training and validating the deep learning model and another part was used for testing. In the part for model training and validation, the images of 100 HCs (85 female) with an average age of 22.7 ± 2.1 years were collected. In the part for model testing, images of 30 TN subjects (19 female) with an average age of 64.0 ± 12.9 years were collected. For the model validation with independent dataset, 67 TN subjects (47 female) with an average age of 57 ± 11.2 years were collected. Informed consent was obtained from all participants prior to their involvement in the study, and the Institutional Review Board of Chang Gung Memorial Hospital approved the study (Institutional Review Board (IRB) Nos. 201901984B0 and 202200372B0).

All TN and HC subjects underwent MRI using a 3-T Siemens Verio MRI system (Siemens Medical System, Erlangen, Germany) equipped with a 32-channel head coil. For model training and testing, 3D magnetization-prepared rapid gradient-echo (MP-RAGE) anatomical images were acquired using a gradient echo sequence. The image resolution was 0.86 × 0.86 × 1 mm^3^ for the HC and 0.45 × 0.45 × 0.9 mm^3^ for TN. The ground-truth labeling for HC was manually assigned by a senior radiologist. For TN, the cisternal segment of the trigeminal nerve was manually labeled on the images by three radiologists, and the final ground-truth labeling was made based on the consensus of the three radiologists. All radiologists are specialists in neuroradiology with 5–20 years of experience in MRI interpretation. Besides, since we incorporated two datasets with different resolutions, we resampled all images to a uniform spatial resolution of 0.7 × 0.7 × 0.7 mm using nearest-neighbor interpolation. This interpolation method was selected as it is best suited for binary images such as trigeminal nerve mask.

### 2.2. Deep Learning Model

**Figure 1:**
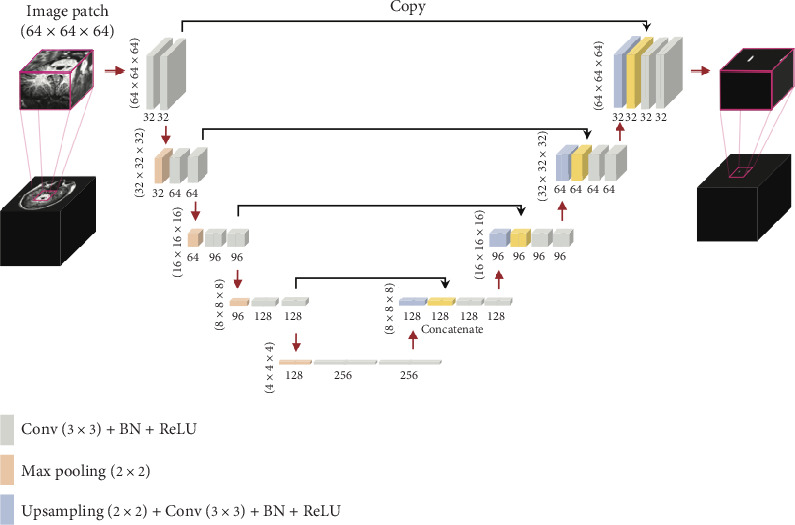
3D U-Net architecture. The 3D U-Net architecture can be visualized as a series of square-shaped feature maps, with each cross-section represented by a box. In the lower left corner of each box, the dimensions of the map are shown, while the number of channels is indicated below those figures. The box on the far left depicts a 64 × 64 × 64 normalized MRI image, derived from the original MRI map. On the far right, we see the prediction of a binary ring mask. Red arrows stand for operations defined by the color-coded box, whereas black arrows denote the copy of skip connections. The terms “Conv,” “BN,” and “ReLU,” respectively, signify convolution, batch normalization, and the rectified linear unit.

Figure 1 presents the 3D U-Net framework [9]. It consists of a contracting path with 32 feature maps in the initial convolutional block, followed by 64, 96, 128, and 256 in the subsequent blocks. During U-Net training, voxels belonging to the trigeminal nerve were assigned a label of 1, while other voxels (background) were labeled 0. The network implementation was carried out using Keras [10] with TensorFlow [11] with an initial learning rate of 1e^−3^and a batch size of 16. The Adam [12] optimizer was utilized, with all parameter gradients clipped to a maximum norm of 1. To address the class-imbalance issue during optimization [13], the Dice coefficient loss [14] was employed as proposed by Ronneberger, Fischer, and Brox [15]. During training, 64 × 64 × 64 sized patches were randomly cropped from all directions as the input, with the patch size optimized for coverage of the bilateral trigeminal nerves while minimizing computational requirements. During inference, extracted and overlapped patches with a 16 × 16 × 16 stride were fed into the trained model. To produce the final output, overlapped predictions were averaged and subsequently resampled to the original resolution via nearest-neighbor interpolation.

### 2.3. Data Augmentation

**Figure 2:**
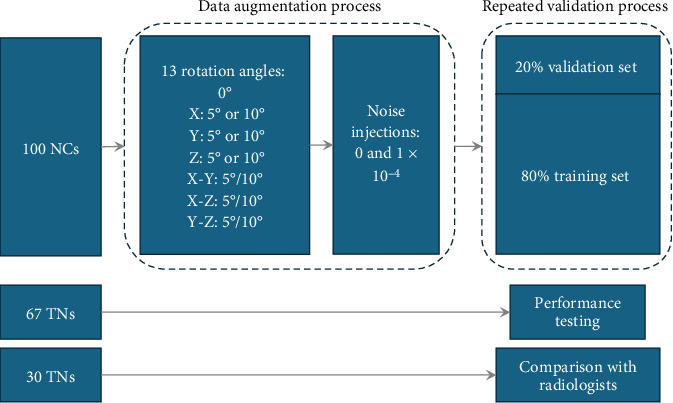
Flowchart illustrating the data augmentation and repeated validation processes. Data augmentation includes 13 types of image rotations: 0°, *x*-axis rotation of 5° or 10°, *y*-axis rotation of 5° or 10°, *z*-axis rotation of 5° or 10°, combined *X*–*Y* rotation of 5 or 10°, combined *X*–*Z* rotation of 5° or 10°, and combined *Y*–*Z* rotation of 5° or 10°. Gaussian noise injections with variances of 0 and 1 × 10^–4^ were also applied. The repeated validation process involved splitting the dataset into 80% training and 20% validation, repeated five times, to ensure robustness in training performance. The 67 TN subjects were used to test the segmentation performance of the 3D U-Net, and a final comparison was made with three radiologists using 30 TN subjects.

We utilized the conventional data augmentation [16] to improve training performance due to limited size of the training data and improve the model generalizability and robustness [17, 18], which includes image rotations [19] and noise injection [20] (Figure 2). Specifically, for each T1 MRI image from the normal control (NC) dataset, we carried out image rotations along the *x*-axis, *y*-axis, *z*-axis, *x*- and *y*-axes, *x*- and *z*-axes, and *y*- and *z*-axes with increments of 5° and 10°. Then, we added Gaussian white noise in all normalized images with a variance of 1 × 10^–4^ (Figure S1). After data augmentation, we obtain 2600 images of HC. Examples of noise injection and signal-to-noise ratio (SNR) calculation is shown in the supporting information, Figure S1.

### 2.4. U-Net Model Training

To demonstrate the robustness and broad applicability of our proposed model for both HC and TN groups, we first formed a training dataset by randomly selecting 80% of the images in the HC (2080 images) and reserving the remaining 20% (520 images) for final performance testing. During the model's training phase, we randomly chose 80% of the data (1664 images) from the training set. The remaining 20% of the data, which accounts for 416 images, was utilized to validate the trained U-Net model. To mitigate any potential bias that might arise from data partitioning, this training–validation cycle was repeated five times. For each U-Net algorithm, the U-Net model with the highest averaged validation accuracy was chosen as the final model for testing in TNs. The performance validation results within the training process are shown in the supporting information, Figure S2.

### 2.5. Evaluation Methods

To assess the segmentation performance of the 3D U-Net quantitatively in both NC and TN groups, a clinical radiologist created the manual trigeminal mask for the NC group, while three clinical radiologists generated the mask for the TN group. Before resampling the data to 0.7 × 0.7 × 0.7 mm for 3D U-Net training, manual segmentation was conducted at the original MRI resolution. The evaluation metrics included the following: (1) volumetric overlap assessments using Dice, which measures the similarity of two samples; (2) Jaccard index, an alternative similarity measure for two sets, addressing instances where the Dice coefficient does not meet the triangle inequality condition; (3) positive predictive value (PPV), illustrating the ratio of true positive predictions; (4) sensitivity (SEN), reflecting the proportion of true positives in the manual delineation process; (5) center-of-mass distance (CMD), determining the Euclidean distance between the centers of mass for two sample sets; and (6) surface distance evaluation employing the Hausdorff distance, which quantifies the separation between two sample sets. The following definitions were used for each: Dice = 2(|*A*∩*B*|)/(|*A*| + |*B*|), Jaccard = (|*A*∩*B*|)/(|*A* ∪ *B*|), PPV = (|*A*∩*B*|)/*B*, SEN = (|*A*∩*B*|)/*A*, and Hausdorff = max{*h*(*A*, *B*), *h*(*B*, *A*)} and $h\left(A,B\right)=\underset{a\in A}{\max}\left\{\begin{array}{c} 0\\ \min\\ b\in B \end{array}d\left(a,b\right)\right\}$, where *A* represents the voxel set of the manually outlined volume, and *B* represents the voxel set of the predicted volume. The term *d*(*a*, *b*) is the Euclidian distance between *a* and *b*. To prevent issues arising from nonuniform data sampling, the Hausdorff distance was calculated only in-plane. The largest Hausdorff distance (i.e., the most significant discrepancy) across slices for each subject was then used for comparative analysis. Better performance is indicated by higher values of Dice, Jaccard, PPV, and SEN and lower Hausdorff values. The computational time for the 3D U-Net method was measured on a Linux-based system (Red Hat Enterprise Linux Server release 7.4 (Maipo)) with an Intel E5-2680 v3 processor (2.50 GHz, 256-GB RAM). It is important to note that the preprocessing steps, including signal normalization and image resampling, were not factored into the computation time. For statistical comparisons among different methods, paired *t*-tests were applied, whereas two-sample *t*-tests were used to contrast images with low and high SNRs in the 3D U-Net. Statistical significance was determined at the alpha level (*p* < 0.05).

### 2.6. Validation on Independent Dataset

To further evaluate the generalizability of our model, we incorporated an independent test cohort consisting of 67 TN subjects. These subjects underwent the same MRI scanning protocols as the original cohort. The primary purpose of incorporating these subjects was to assess the model's ability to maintain segmentation accuracy across a more diverse set of individuals, particularly to validate the performance in a scenario representative of clinical usage. This dataset was used exclusively for testing and was not part of the original training or validation sets, thereby providing an unbiased evaluation of the model's robustness.

### 2.7. Comparison of the Segmentation Performance With Radiologists

To assess the effectiveness of the proposed 3D U-Net algorithm for trigeminal nerve segmentation, we compared its performance with that of three radiologists (Rater 1, Rater 2, and Rater 3), each with 5–15 years of experience in diagnostic neuroradiology. A total of 30 cases of TN, independent from the training, testing, and validation datasets, were included in this analysis. Performance metrics, including the Dice coefficient, Jaccard index, PPV, and SEN, were used to evaluate segmentation accuracy. Additionally, CMD and Hausdorff distance were calculated to assess spatial agreement with the ground truth.

## 3. Results

### 3.1. Segmentation Performance of Healthy Control

**Figure 3:**
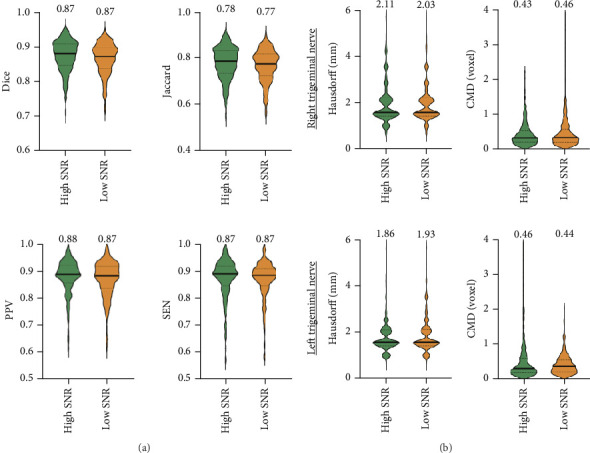
Trigeminal nerve segmentation performance metrics for 3D U-Net on original HC images (high SNR) and HC images with white noise addition (low SNR). The average values of (a) similarity metrics (Dice, Jaccard, SEN, and PPV) and (b) distance metrics (CMD and Hausdorff distance) are shown above each bar. We estimate that the two-sample t-tests were used for statistical comparison among images with low and high SNR.

Figure 1 illustrates the performance of our trained 3D U-Net algorithm for trigeminal nerve identification in the HC. Across all measures, 3D U-Net showed outstanding trigeminal nerve segmentation performance (Dice, PPV, and SEN > 0.85) in both low- and high-SNR images (Figure 3). Notably, although the images with white noise showed significant lower SNR compared to original images (Figure S1), the 3D U-Net approach produces ideal results with Dice > 0.85 in both low- and high-SNR images, and no significant difference was found in all the segmentation performance metrics (Dice, Jaccard, PPV, and SEN). Furthermore, for evaluating the spatial overlap of 3D U-Net, the low averaged Hausdorff distance (2.07 in the right and 1.90 in the left trigeminal nerve) and averaged CMD (0.45 in both right and left trigeminal nerves) of low- and high-SNR images further indicate its best match segmentation, which represents that the predicted segmentation is a reliable representation of the ground truth. Together, these results suggest that 3D U-Net is a reliable and reproducible approach for trigeminal nerve segmentation in NC.

### 3.2. Generalizability Performance on Independent Cohort

**Figure 4:**
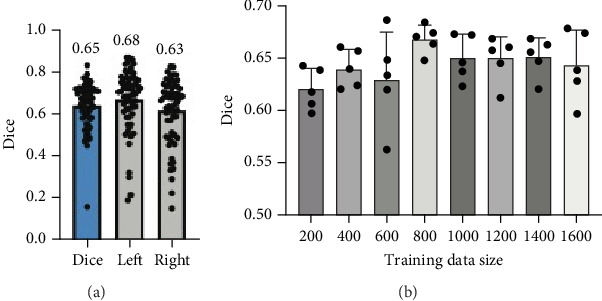
Segmentation performance of 3D U-Net on (a) an independent test dataset of 67 subjects and (b) across model-validating sample sizes. The 3D U-Net was trained from randomly selected subgroups from the training dataset, with each random selection repeated five times. In the training process, we randomly selected 200–1600 training images (out of the total 1664 used for training) in increments of 200 images from the total 2600 training images, repeating this random selection five times for each increment. The results show average accuracy stabilizing after the training data size reaches 800 subjects, indicating reliable model performance with increasing data.

The performance of the model on the new dataset consisting of 67 subjects demonstrated consistent accuracy in the segmentation of the trigeminal nerve. Specifically, the average Dice coefficient was 0.65, which was comparable to the results obtained with the original dataset, confirming the model's robustness (Figure 4(a)). Furthermore, segmentation accuracy was assessed separately for the left and right sides of the brain (average Dice coefficient for left: 0.68, right: 0.63). These findings support the model's ability to generalize well beyond the initial dataset, maintaining stable performance even when the size of training data was varied, as shown in Figure 4(b). The model's accuracy appeared to plateau after the training data size reached 800 subjects, remaining consistent around an average Dice coefficient of 0.65.

### 3.3. Comparison of the Segmentation Performance With Radiologists

**Figure 5:**
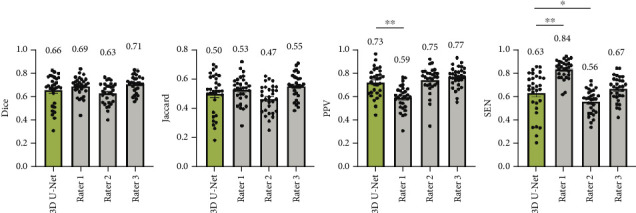
Trigeminal nerve segmentation performance metrics for 3D U-Net and three radiologists (Rater 1, Rater 2, and Rater 3) on TNs. The mean value is displayed above each bar. For statistical comparison between the 3D U-Net and three different raters, two-tailed paired *t*-tests were implemented (⁣^∗^*p* < 0.05 and ⁣^∗∗^*p* < 0.01).

To evaluate the performance of our 3D U-Net algorithm in segmenting the trigeminal nerve, we included 30 cases of TN.  Figure 5 provides a comparison between the 3D U-Net algorithm and three radiologists (Rater 1, Rater 2, and Rater 3) with respect to their segmentation accuracy for trigeminal nerve identification. Key performance metrics, including Dice coefficient, Jaccard index, positive predictive value (PPV), and SEN, were used to assess the effectiveness of each method.

**Figure 6:**
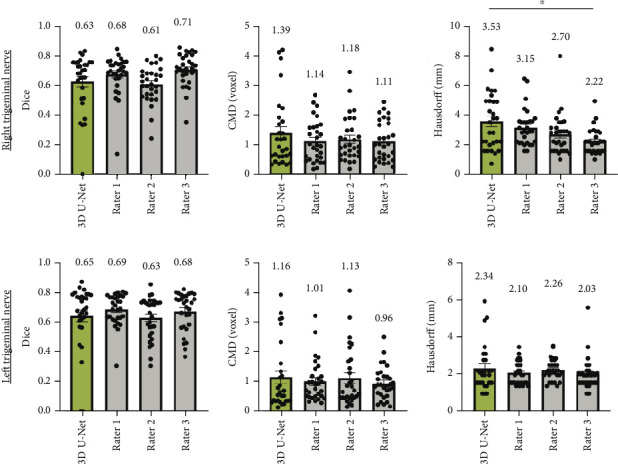
Bilateral trigeminal nerve spatial overlap measurements for 3D U-Net and three radiologists (Rater 1, Rater 2, and Rater 3) on TNs. The mean value is displayed above each bar. For statistical comparison between the 3D U-Net and three different raters, two-tailed paired *t*-tests were implemented (⁣^∗^*p* < 0.05).

The 3D U-Net model demonstrated comparable segmentation accuracy to the radiologists, showing no significant differences in Dice and Jaccard scores across all raters. Notably, Rater 2 achieved a high PPV (0.75, *σ* = 0.113) but a low SEN (0.56, *σ* = 0.102), indicating undersegmentation of the trigeminal nerve. Conversely, Rater 1 exhibited a lower PPV (0.59, *σ* =0.103) and a higher SEN (0.84, *σ* = 0.080), suggesting an overestimation of the nerve region. Furthermore, no significant differences in Hausdorff distance or CMD were observed between the 3D U-Net model and the radiologists, underscoring the algorithm's ability to achieve spatial accuracy comparable to that of human raters (see Figure 6).

Additionally, the 3D U-Net model demonstrated efficiency, with an average computation time of 2.4 min per case (*σ* = 0.002 min), highlighting its potential as a viable alternative to manual segmentation by radiologists.

**Figure 7:**
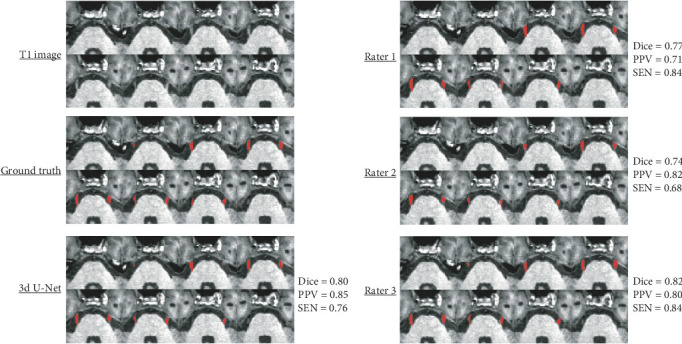
The best segmentation comparisons for TN images are presented, chosen based on the maximum average Dice score. This score is noted to the right of the brain map and is determined by averaging the results from four different methods (3D U-Net, Rater 1, Rater 2, and Rater 3).

**Figure 8:**
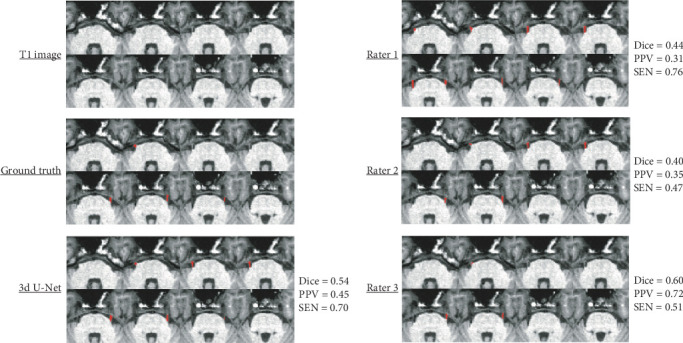
The worst segmentation comparisons for TN images are presented, chosen based on the maximum average Dice score. This score is noted to the right of the brain map and is determined by averaging the results from four different methods (3D U-Net, Rater 1, Rater 2, and Rater 3).

Figures 7 and 8 showcase the best and worst cases, respectively, in terms of Dice score among all methods (3D U-Net, Rater 1, Rater 2, and Rater 3). In the best-case scenario, both the 3D U-Net and Rater 3 achieved high segmentation accuracy (Dice > 0.80), with Rater 1 and Rater 2 also performing well (Dice = 0.77 and 0.74, respectively). However, Rater 1 showed a lower PPV (0.71) and Rater 2 showed lower SEN (0.68). In the worst-case scenario, only the 3D U-Net and Rater 3 maintained a Dice score above 0.5, while Rater 1 and Rater 2 struggled to segment the trigeminal nerve accurately (Dice < 0.5).

### 3.4. Effect of Noise on Segmentation Accuracy

**Figure 9:**
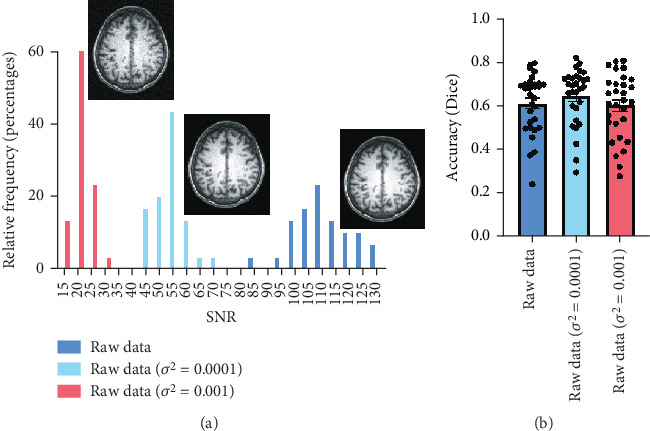
Effect of noise levels on SNR distribution and segmentation accuracy. (a) SNR distributions for the raw data and datasets with added Gaussian noise (*σ*^2^ = 0.0001 and *σ*^2^ = 0.001) are shown. (b) Segmentation accuracy measured by the Dice coefficient for the raw data and noisy datasets. Despite the added noise, no significant difference in accuracy was observed, demonstrating the robustness of the model.

To evaluate the impact of noise levels on segmentation accuracy, we conducted experiments using different Gaussian noise variances. As shown in Figure 9(a), the SNR distribution changes significantly with the introduction of different levels of noise. Specifically, the addition of Gaussian noise with variances of *σ*^2^ = 0.0001 and *σ*^2^ = 0.001 resulted in a shift of the SNR values towards lower ranges. Despite this, no significant difference in accuracy was found.  Figure 9(b) shows the segmentation accuracy, as quantified by the Dice coefficient. The average Dice coefficient changed from 0.62 ± 0.13 for the raw data to 0.65 ± 0.13 and 0.60 ± 0.15 for the datasets with *σ*^2^ = 0.0001 and *σ*^2^ = 0.001, respectively. This indicates that the model maintained its robustness even with added noise, as no significant difference in accuracy was observed.

## 4. Discussion

We developed a deep learning–based automatic segmentation model for the trigeminal nerve that demonstrates satisfactory performance in both HC and TN patients. The proposed framework, which uses 3D U-Net, is robust, accurate, and automatic in extracting trigeminal nerve from MR images. To the best of our knowledge, this is the first fully automated segmentation method for the trigeminal nerve in anatomic MRI.

MRI of the brain is now the standard method used to rule out any secondary causes of TN [21]. Accurate and detailed trigeminal MRI scan sequences are necessary for detecting any trigeminal neurovascular conflicts, identifying the vascular structure causing the conflict, and determining the severity of compression. Currently, the protocol should include a combination of three high-resolution sequences [22]: first, a 3D T2-weighted sequence (fast imaging employing steady state (FIESTA), driven equilibrium (DRIVE), and constructive interference in steady state (CISS)) which provides excellent contrast between the cerebrospinal fluid (high signal intensity) and neurovascular structures (low signal intensity) producing high-performance cisternography; second, a time-of-flight MR-angiography which provides good visualization of the arteries in contrasting with the cerebrospinal fluid; and third, a 3D T1-weighted (T1W) gadolinium sequence which allows the visualization of nerves in relation to cerebrospinal fluid and shows both arteries and veins in high signal and to exclude any enhancing lesion that may cause secondary TN [23]. Lin et al. recently introduced a 3D convolutional neural network–based automatic segmentation method for the trigeminal nerve in MR angiography [24]. While MR angiography provides excellent visualization of nearby arteries that may cause neurovascular conflicts, the trigeminal nerve is of intermediate signal on time-of-flight MR-angiography, with lower contrast with the cerebrospinal fluid compared to the 3D T1W or T2-weighted sequence. Therefore, segmentation of the trigeminal nerve in anatomic images can provide higher accuracy, especially in patients with severe nerve distortion or atrophy [25]. Additionally, the 3D T1W sequence is more commonly used in clinical MRI compared to the 3D T2-weighted sequence or MR angiography. An automatic segmentation tool based on T1W anatomic imaging has the potential to detect nerve status more practicable in the general population. Notably, due to the relatively small size of cranial nerves, routine spin-echo or gradient-echo T1W images with thick slice thickness are not feasible for segmentation. We recommend using 3D T1W imaging with submillimeter slice thickness to achieve the necessary resolution for precise segmentation of the trigeminal nerve.

The 3D U-Net architecture we propose provides a dependable and accurate approach for segmenting the trigeminal nerve in T1W anatomical imaging. This is due to its ability to directly investigate and learn hierarchical features from the training dataset. The 3D U-Net encoder employs 3D convolution followed by 3D max pooling to extract features, while the decoder utilizes 3D upsampling to reconstruct annotated images [26]. By merging location information from the downsampling path with contextual information from the upsampling path, the 3D U-Net achieves a balance of localization and contextualization for reliable segmentation prediction [15]. Additionally, the model's ability to utilize interslice contextual information allows it to extract more intricate features from the training dataset, further enhancing its segmentation accuracy. To improve the model's performance and increase its generalizability and robustness given the limited size of the training data, we utilized conventional data augmentation [16]. We examined its accuracy on the HC with noise injection and found that 3D U-Net has a noise-resistant capacity. The results highlight the stability and robustness of 3D U-Net in segmenting the trigeminal nerve in the HC with noise injection, even when the image SNR is halved. This demonstrates that 3D U-Net can handle MRI data across a wide range of SNR quality. Overall, the 3D U-Net architecture provides a reliable and precise method for segmenting the trigeminal nerve in T1W anatomic imaging, and its noise-resistant capacity makes it a valuable tool for handling MRI data in various SNR conditions.

Accurate segmentation of the trigeminal nerve is crucial in diagnosing and determining the severity of TN symptoms and response to treatment, particularly in patients with neurovascular contact [25, 27]. The displacement or atrophy of the trigeminal nerve caused by neurovascular contact is highly associated with TN symptoms [1, 27]. Furthermore, the volume of the trigeminal nerve has been shown to be associated with recurrence following radiofrequency rhizotomy [28], making it even more important to accurately segment the nerve in TN patients undergoing this treatment. Despite the benefits of accurate segmentation, it can be difficult to identify the trigeminal nerve even on high-resolution imaging, particularly in cases of severe distortion or atrophy. Our segmentation model exhibits good performance in HC images with a Dice score of over 0.85 but has a relatively lower performance in TN images. However, the performance is comparable to that of participating radiologists. This may be attributed to the difficulty in accurately defining the cisternal or preganglionic segment of the trigeminal nerve, especially at critical regions such as the root entry zone and the porus trigeminus where the nerve enters Meckel's cave. In a single image voxel, nerve fibers, adjacent vascular structures, and cerebrospinal fluid are often mixed, even in high-resolution images obtained using modern MR scanners. The presence of vascular compression may cause the nerve to be distorted resulting in a redundant trajectory and a thinner nerve at the site of compression. In cases of severe nerve atrophy due to long-term vascular compression, the trigeminal nerve may not be fully delineated or only be partially visible within a few image voxels. The variability in segmentation results between readers and the time-consuming nature of manual segmentation can result in a lack of consistency. This highlights the need for more precise and automated segmentation methods that can aid in the diagnosis and treatment of TN.

The development of automatic segmentation methods for the trigeminal nerve has opened a wide range of possibilities for the future application of deep learning in the diagnosis and treatment of TN. One potential application is the classification of TN subtypes, such as distinguishing between classical and idiopathic TN. This would allow for more targeted and effective treatment options for patients with TN. Another potential use of automatic segmentation is for intraoperative imaging for navigation of microvascular decompression and radiofrequency ablation. With accurate and automated real-time segmentation, surgeons can more precisely locate and treat the source of compression, resulting in better patient outcomes [4, 29, 30]. Furthermore, automatic segmentation can aid in patient selection for determining appropriate treatment options. By accurately measuring the volume and status of the trigeminal nerve, clinicians can better predict which patients will respond well to certain treatments and which may require more aggressive interventions. Finally, the use of deep learning in automatic segmentation may also allow for the prediction of treatment response, helping clinicians to better tailor treatment plans to individual patients. Overall, the future of automatic segmentation in TN holds great promise for improving diagnosis, treatment, and outcomes for patients with this challenging condition.

## 5. Conclusion

In conclusion, the proposed method of automatic segmentation of the cisternal segment of the trigeminal nerve using deep learning demonstrates a high degree of accuracy, SEN, and specificity. This promising approach offers an automated and efficient alternative to manual segmentation, which can be subject to inter- and intrarater variability and time-consuming. Furthermore, we believe it has the potential to be applied in clinical settings, assisting in the diagnosis and treatment planning of TN.

## Data Availability

The data that support the findings of this study are available from Chang Gung Memorial Hospital, but restrictions apply to the availability of these data, which were used under license for the current study, and so are not publicly available. Data are however available from the authors upon reasonable request and with permission of Chang Gung Memorial Hospital.

## Supporting Information

Supporting Information Additional supporting information can be found online in the Supporting Information section. The supporting information included the noise injection and performance validation within the training process.
