# Supplementary material for: Automatic Segmentation of the Cisternal Segment of Trigeminal Nerve on MRI Using Deep Learning
Source: Int J Biomed Imaging. 2025 Feb 16;2025:6694599. doi: 10.1155/ijbi/6694599 (PMC11847612; doi:10.1155/ijbi/6694599)
Supplement: Supporting Information — Additional supporting information can be found online in the Supporting Information section. The supporting information included the noise injection and performance validation within the training process. [file 6694599.f1.PDF]

Supplementary materials:

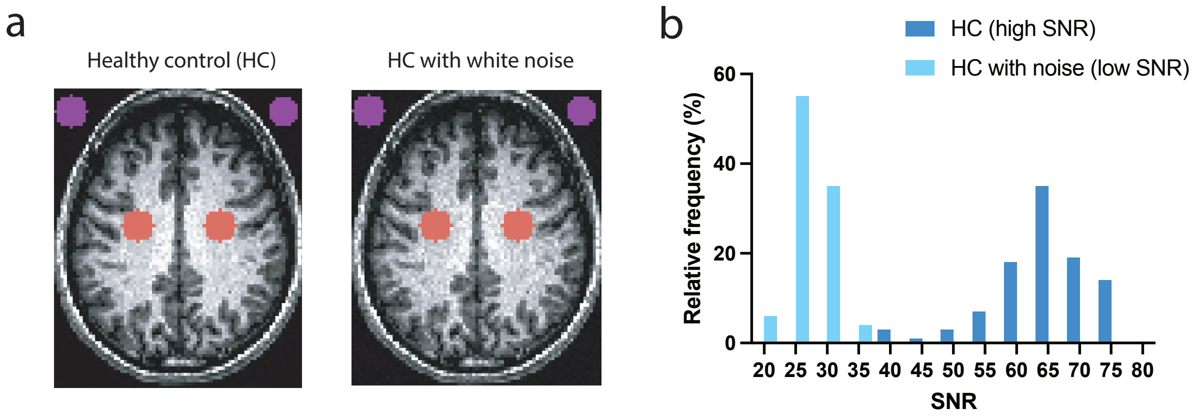

Figure S1. Example of noise injection and SNR calculation. a) One example image from healthy control and the image after noise injection. The SNR was estimated to represent the image noise level by calculation the ratio of the signal intensity in the area of interest (orange area) to that of the background (purple area). The diameter of area of interest = 10 mm. b) the distribution of SNR among original HC images and adding white noise.

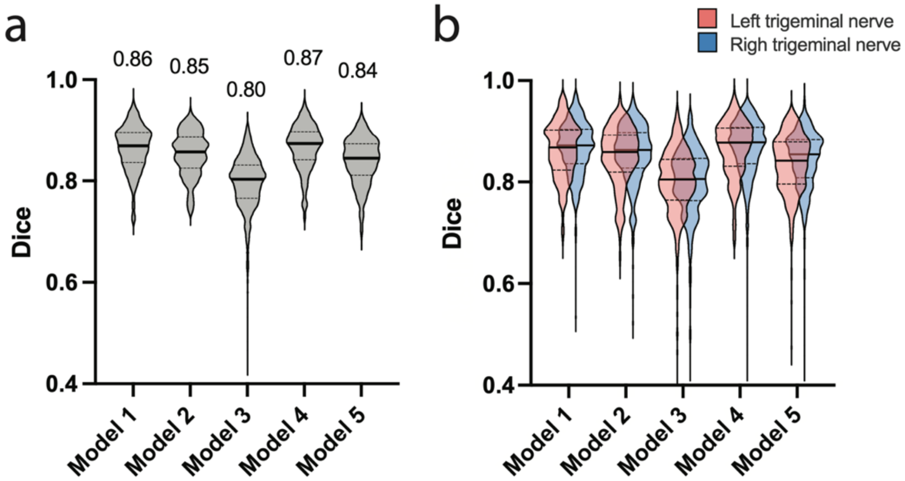

Figure S2. Performance validation results within the training process. a) In the training process, we randomly selected 80% of the HC data (1664 images) from the training dataset. The remaining 20% of the HC data (416 images) from the training dataset was used for validating the 3D U-Net model. We repeated this training validation process five times to avoid randomness bias in data splitting. The 3D U-Net model with highest averaged validation accuracy was then used as the final model for testing. b) The segmentation performance was estimated in the left and right trigeminal nerve separately, and no significant difference was found in each model.
